# The Role of Deubiquitinating Enzymes in the Various Forms of Autophagy

**DOI:** 10.3390/ijms21124196

**Published:** 2020-06-12

**Authors:** Tamás Csizmadia, Péter Lőw

**Affiliations:** Department of Anatomy, Cell and Developmental Biology, Eötvös Loránd University, 1117 Budapest, Hungary; tamas.csizmadia@ttk.elte.hu

**Keywords:** cargo degradation, DUB, lysosome, ubiquitin, vesicle fusion

## Abstract

Deubiquitinating enzymes (DUBs) have an essential role in several cell biological processes via removing the various ubiquitin patterns as posttranslational modification forms from the target proteins. These enzymes also contribute to the normal cytoplasmic ubiquitin pool during the recycling of this molecule. Autophagy, a summary name of the lysosome dependent self-degradative processes, is necessary for maintaining normal cellular homeostatic equilibrium. Numerous forms of autophagy are known depending on how the cellular self-material is delivered into the lysosomal lumen. In this review we focus on the colorful role of DUBs in autophagic processes and discuss the mechanistic contribution of these molecules to normal cellular homeostasis via the possible regulation forms of autophagic mechanisms.

## 1. Introduction

The homeostatic equilibrium of the eukaryotic cells is maintained by several cell biological processes. The cellular self-digestive mechanisms have a main contribution in the fast-cellular response to the changeable environment. The essential function of these mechanisms is to degrade several types of cellular components such as a specified part of the cytoplasm, signaling molecules, proteins and cell organelles. Beside the cellular material breakdown there is another importance of the self-digestive mechanisms in the cellular life: the eukaryotic cells use these mechanisms to recycle the essential molecules of the degraded material into the cytoplasm. The unnecessary or injured components of cells can be degraded by lysosome dependent (autophagy) and independent (Ubiquitin-Proteasome System—UPS) pathways [1,2,3,4]. Importantly, autophagy is responsible for the breakdown of the significant cytoplasmic material (macromolecules) and cell organelles such as mitochondria, peroxisomes, ER, part of the nucleus, secretory granules and damaged lysosomes [1,2,5]. In contrast the UPS is only capable of removing and recycling short half-life proteins from the cytoplasm, which control several signaling pathways [3,4,6]. Unfortunately, various types of disorders are connected to the reduced function of these self-degradative mechanisms such as cancer, diabetes, accelerated aging, fatty liver disease (FLD) and infectious, neurodegenerative and vascular diseases [7,8,9]. Notably, autophagy and UPS work together and complement each other for the effective degradation of cellular components which leads to normal homeostatic equilibrium during various internal or external effects [3,10,11]. Furthermore, ubiquitin, as a frequent posttranslational modifying molecule, also has a role in proteasome independent mechanisms such as epigenetic regulation, DNA damage response, mitophagy, cell cycle control, diverse signaling pathways and intracellular vesicular and protein trafficking mechanisms [12,13].

In this review we summarize and discuss the main connection points of autophagic degradation pathways and ubiquitin signaling, especially the role of deubiquitinating enzymes in several lysosome-dependent self-degradative processes.

## 2. The Phenomenon of Autophagy

**Figure 1:**
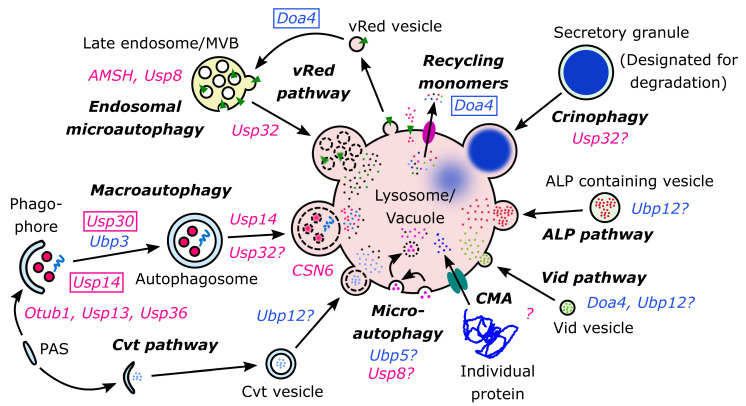
Summary of autophagic and related recycling processes with the connecting deubiquitinases (DUBs) in the eukaryotic cells. The indicated DUBs occur in metazoan (marked with magenta) and yeast (marked with blue) cells. The DUBs framed have negative regulation effects in the relevant autophagic processes.

During autophagy the lysosomal compartment, as the terminal place of degradation, has a central role in digestion and recycling mechanisms. There are four major types of autophagic processes depending on how cellular materials enter the lysosomal lumen: macroautophagy, microautophagy, chaperon-mediated autophagy and crinophagy [1,2,5]. Moreover, there are several “exotic” autophagy-like processes, which were also identified in model systems of yeast and *Drosophila* cells (Figure 1) [14,15,16,17].

### 2.1. Macroautophagy

The best-known lysosome-dependent self-degradative process is macroautophagy, which is the focus of several biomedical research studies. The main hallmark of this process is the formation of a phagophore cistern, by which the cytoplasmic components are separated and captured into double membrane bound autophagosomes. These compartments transport their cargo to the acidic lysosomal lumen for fast degradation of cytoplasmic molecules and organelles [18]. Autophagosomes were detected for the first time in 1959 by Novikoff in hydronephrotic renal tissue [19]. Autophagosomes shape at special sites of the cytoplasm and they are created by the coordinated action of the evolutionary conserved Atg (Autophagy related) proteins, which form distinct protein complexes. The first set of *atg* genes were identified and characterized in yeast (*Saccharomyces cerevisiae*) by Yoshinori Ohsumi and his research group [20]. This discovery led to his Nobel Prize in Physiology or Medicine in 2016. The successful degradation of the autophagosome transported cytoplasmic cargo requires the direct fusion of these compartments with endosomes and lysosomes. There are main factors, which are necessary for this membrane fusion step: the tethering complex *HOPS* (homotypic fusion and vacuole protein sorting) modulated by normal expression of the Vps8 protein, the small GTPases Rab2, Rab7 (and its partners Mon1/Ccz1 and Plekhm1), Arl8 and the specific SNARE (soluble NSF attachment protein receptor) proteins including autophagosomal syntaxin 17 (*Syx17*) and its binding partners Snap29 and Vamp8 (Vamp7 in *Drosophila*) [21,22,23,24,25,26,27,28,29,30,31]. As a result of autophagosome-lysosome fusion, both the transported cytoplasmic cargo and the inner membrane of the autophagosome are digested, while the outer autophagosomal membrane blends into the membrane of the resulting secondary lysosome (autolysosome). The recycling of the monomers released from cargo degradation requires special efflux transporter proteins (permeases), which are localized in the membrane of lysosomes [32]. This recycling step allows the monomers to be recycled into the cytoplasm where these fuel biosynthetic and energy production processes.

### 2.2. Microautophagy and Endosomal Microautophagy

The second form of autophagic processes is microautophagy which is mediated—in plants and fungi by vacuolar action—during the direct engulfment of the surrounding cytoplasmic cargo [33]. This cytosolic material is trapped in the lysosome/vacuole by a non-selective random process of membrane invagination to create small intraluminal vesicles, which are then degraded. Depending on the mechanistic nature of microautophagy, three subtypes of this process are distinguished: 1. microautophagy by lysosomal invagination (this is a classic form), 2. microautophagy through a lysosomal arm-like protrusion (which encompass the cytoplasmic components) and 3. microautophagy by endosomal invagination [34]. Latter, namely endosomal microautophagy is a special form of this process, which typically occurs in metazoans such as *Drosophila melanogaster* and mammals [5,35]. During endosomal microautophagy the incorporation of the cytoplasmic material is conducted by late endosomes. Importantly, microautophagy depends on the activity of ESCRT (endosomal sorting complex required for transport) proteins that also mediate the sorting of internalized receptor ligand complexes in multivesicular bodies [35,36]. During microautophagy the late endosomal/lysosomal or vacuolar surface proteins interact with special organelle proteins for their selective degradation. Moreover, the heat shock cognate 70 kDa protein (HSC70) as a cytosolic chaperone is also necessary for protein degradation via microautophagy [16,37]. This process may resemble endocytosis a little, but the topology of microautophagy shows an opposite direction, because the lysosomal lumen is equivalent with the extracellular space. Several types of cellular organelles such as small secretory granules, mitochondria, peroxisomes and even part of the nucleus can be degraded by microautophagy. Even so, the capacity of microautophagic pathway lags behind that of macroautophagy. Importantly, the cellular survival during starvation or nitrogen deprivation conditions requires the microautophagic activity, which is also necessary for maintaining cellular homeostasis [1,2,5,35,37,38].

### 2.3. Chaperone-Mediated Autophagy (CMA)

The special lysosome dependent self-degradative pathway is chaperone-mediated autophagy (CMA), which operates without the digestion of biological membranes. It involves the selective degradation of KFERQ-like motif-bearing proteins, which are normally hidden within potential target proteins [39]. The cytosolic chaperone HSC70 and its cochaperones, such as the carboxyl terminus of HSC70-interacting protein (CHIP), the heat shock protein 70 and 90 (HSP70–HSP90) organizing protein (HOP), and HSP40 also known as DNABJ1, are all required for the delivering of the old or damaged proteins to the lysosomes. Moreover, the exposer of the earlier mentioned KFERQ-like amino acid sequence on the surface of these proteins is necessary for the recognition and lysosomal degradation of these abnormal proteins. The target protein internalizes in lysosomes via the channel formed by the lysosome-associated membrane protein type 2A (LAMP2A). Finally, these captured proteins are rapidly degraded by lysosomal hydrolases [5,39,40]. CMA as a protein quality control mechanism rapidly degrades damaged and oxidized proteins which are generated by stress conditions. This process is also necessary for amino acid recycling through the intensive elimination of low-quality proteins from the cytoplasm during starved conditions [39,40]. Moreover, CMA also modulates multiple cellular pathways depending on the nature of the protein substrate that is degraded. Interestingly, CMA, as a selective remodeling pathway of the proteome, is also required for the regulation of cell cycle, lipid and carbohydrate metabolism, transcriptional programs and immune responses [40]. It is important to note that there are no data about deubiquitinating enzymes’ (DUBs) contribution for this type of autophagy.

### 2.4. Secretory Granule-Lysosome Fusion (Crinophagy)

Crinophagy is the least known and mysterious type of autophagy during which the unnecessary, obsolete or damaged secretory granules instead of their exocytosis directly fuse with late endosomes or lysosomes. This process leads to the fast degradation of unused secretory cargo and the released monomers able to recycle into the cytoplasm through lysosomal permeases [1,2,41]. The phenomenon of crinophagy was first identified in 1966 by Smith and Farquhar per the examination of the lysosomal activity in the anterior pituitary gland of female rats during the lactation process [42]. The molecular background of secretory granule-lysosome fusion was unknown and unclear for a long time. The first molecule that directly regulates crinophagy was discovered in 2018 by the examination of developmentally programmed secretory granule degradation in *Drosophila* late larval salivary gland cells [43]. Our laboratory identified that crinophagy uses similar molecules to the late step of macroautophagy (autophagosome-lysosome fusion): the tethering complex HOPS (modulated by normal Vps8 expression), the small GTP-ases Rab2, Rab7 (and its binding partner Plekhm1), Arl8 and specific SNARE proteins Snap29 Qbc-SNARE and Vamp7 R-SNARE. There is one difference between the membrane fusion apparat of macroautophagy and crinophagy: the former process uses syntaxin 17 (Syx17), whereas in contrast, secretory granule-lysosome fusion requires syntaxin 13 (Syx13) [30,31,41,43]. It seems that the type of Qa SNAREs may specify what kind of compartment is able to fuse with late endosomes or lysosomes. As a result of secretory granule-lysosome fusion, the transported secretory cargo is fully digested, while the membrane of the secretory granule blends into the membrane of the resulting secondary lysosome (crinosome) [41,43,44]. The human body contains several gland tissues, which all use crinophagy to control the secretory vesicle pool, so this process has huge medical relevance. Crinophagy is upregulated in several disorders (such as acute pancreatitis and type 2 diabetes), so the molecular background, which leads to zymogen and insulin granule degradation, should be explored [45,46,47,48]. However, the main molecules, which coordinate the crinophagic membrane fusion mechanisms, have already been identified, but the transcriptional regulation of this process and the molecular designation of secretory granules for degradation still remains obscure. Our laboratory currently is working to find answers for these interesting and relevant questions using the *Drosophila* salivary gland as a powerful model system [43,49].

### 2.5. Cytoplasm to Vacuole Targeting (Cvt) Pathway

The cytoplasm to vacuole targeting (Cvt) pathway is a selective type of autophagy in yeast. This process, considering its mechanistic nature and molecular components, is very similar to macroautophagy [14]. Proenzymes, (such as the precursor of aminopeptidase 1—preApe1) are synthetized in the cytosol, aggregate with each other (Ape1), and these particles are specifically captured by phagophore like structures. Thereafter, these enzymes are packed into double membrane-bound small autophagosome like vesicles [50]. The core Atg proteins, such as Atg7, Atg8 and Atg9, which control autophagosome assembly and maturation are also necessary for Cvt vesicle formation. Several molecules are required for the recognition of Ape1 particles in the cytoplasm, such as the scaffold protein Atg11 and the receptor molecule Atg19. Finally, the mature Cvt vesicles fuse with the vacuole in a HOPS-, SNARE- and Rab-dependent manner. Importantly, the aim of this mechanism is not the degradation of cargo. The supplied bulk Ape1 becomes active by other hydrolases in the vacuole [14,50,51].

### 2.6. The Vacuole Membrane Protein Recycling and Degradation (vRed) Pathway

Vacuole membrane protein recycling and degradation (vRed) is a recently identified autophagy-like pathway for the rapid elimination of the vacuolar efflux lysine transporter Ypq1 from the vacuolar membrane. It was discovered in 2015 by Li and Emr in lysine starved yeast cells [15]. During this mechanism the unnecessary lysine transporter Ypq1 after its VAcUL-1-depending polyubiquitination is selectively sorted off the vacuolar membrane to the small cytoplasmic (vRed) vesicles. The ESCRT machinery is recruited to these vesicles, and the polyubiquitinated Ypq1 is sorted into the intraluminal vesicles of the multivesicular endosomes. Finally, these compartments are delivered into the vacuole for degradation in a HOPS- and SNARE-dependent manner [15]. Furthermore, similar mechanisms are responsible for the elimination of the vacuolar Zn^2+^ influx transporter Cot1 and Zn^2+^ channel Zrt3 [16]. Thus, these mechanisms are required for the rapid elimination of vacuolar efflux and influx transporters as well as ion channels depending on the actual environmental effects [15,16].

### 2.7. Alkaline Phosphatase (ALP) Pathway

During the alkaline phosphatase (ALP) pathway the resident hydrolases are transported from the Golgi to the vacuole for activation [14]. ALP is synthesized as an inactive precursor enzyme, which contains a C-terminal propeptide sequence. The ALP containing vesicles directly fuse with the vacuole in a HOPS-dependent manner [52,53]. During the activation process the C-terminal propeptide is cleaved off from the ALP protein by Pep4 vacuolar hydrolase. It is important to note that the ALP pathway is similar to the lysosome derived vesicle pathway in metazoans [14,52,53,54].

### 2.8. Vacuole Import and Degradation (Vid) Pathway

The vacuole import and degradation (Vid) pathway is a special autophagic mechanism, which can be found in yeast cells after glucose readdition. This phenomenon was discovered by Chiang and Schekman in 1991 via the examination of glucose starved yeast cells [14,55]. During glucose deprivation yeast cells produce a high amount of gluconeogenic enzymes, such as Fructose 1-6 BisPhosphatase (FBPase), Malate DeHydrogenase 2 (MDH2), Phosphoenol-pyruvate carboxykinase 1 (Pck1) and Isocitrate lyase 1 (Icl1) in the cytoplasm [56]. After glucose readdition to the media, yeast cells degrade these enzymes really quickly in two ways: on one hand gluconeogenic proteins can be polyubiquitinated by the CTLH domain containing E3 ubiquitin ligase complex (Gid—glucose induced degradation) for the proteasomal breakdown of these proteins [57,58]. On the other hand, gluconeogenic enzymes can also be captured into small vesicles (Vid vesicles), then these compartments fuse with the vacuole directly (Vid pathway) [55,59], which leads to the really fast degradation of these obsolete cytoplasmic enzymes.

## 3. Short Overview of the Ubiquitination System

### 3.1. Ubiquitin as a Multiple Importance Posttranslational Modification Molecule

**Figure 2:**
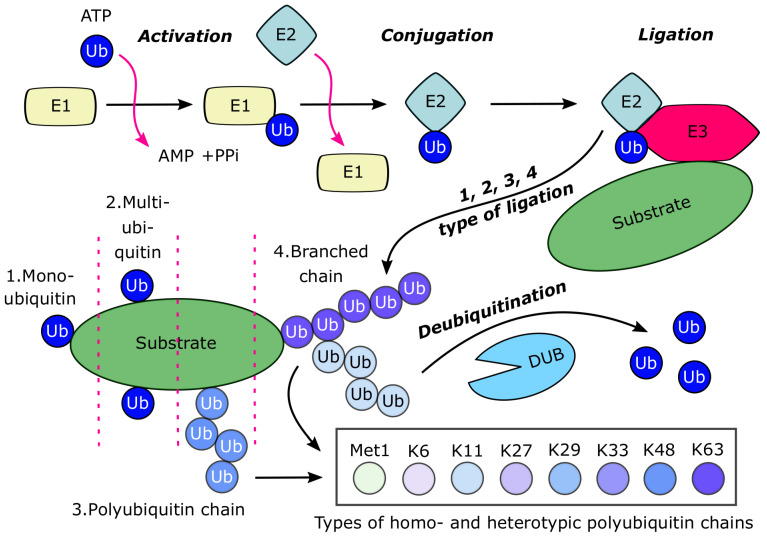
Overview of the ubiquitinating system (Abbreviations: Ub—ubiquitin, E1—ubiquitin activating enzyme, E2—ubiquitin conjugating enzyme, E3—ubiquitin ligase, DUB—deubiquitinase enzyme, Met—methionine, K—lysine).

Ubiquitin, a highly conserved 76 amino acid protein, is expressed in all cell types and is fundamental to various biological functions [60]. Ubiquitin, as a well-known posttranslational modification of several substrates with covalent conjugation, typically connects via Lys residues, although sometimes via Cys, Ser and Thr residues. The induction or reversion of ubiquitination (similar to other posttranslational modifications) is regulated by special enzymes. Usually, there is a single ubiquitin activator enzyme (E1), but there are many species of ubiquitin conjugating enzymes (E2s) and multiple families of E3 ubiquitin ligases or E3 multiprotein complexes in a cell (Figure 2) [60,61,62]. Ubiquitin has several possible molecular sites for various types of posttranslational modifications, such as acetylation, phosphorylation, sumoylation, neddylation and ubiquitination. Interestingly, ubiquitin has seven Lys residues (K6, K11, K27, K29, K33, K48 and K63), all of which can be attached with other ubiquitin molecules to give rise to isopeptide-linked polyubiquitin chains. Moreover, the eighth chain type is the methionine (Met1)-linked or linear chain, which is also generated on ubiquitin by the attaching to the N-terminus of a second ubiquitin (Figure 2) [12,63,64]. Importantly, the type of the polyubiquitin chain conjugated to the substrate can specify the fate of the protein substrate, by which ubiquitination may influence various forms of biological pathways. Approximately, there are 1000 E3 enzymes, which have a role in these colorful substrate ubiquitination processes in mammals [65]. Thus, these types of enzymes play critical roles in the regulation of numerous biological functions via the ubiquitination of the key substrates. The most well-known and understood function of the target substrate ubiquitination is the protein designation for proteasomal degradation with K11- or K48-linked polyubiquitin chains [60,66]. The biological outcomes of the substrate ubiquitination depend on the modification type of the key substrate proteins (Figure 2); therefore, they are not all limited to the proteasomal degradation of substrates [67]. For instance, ubiquitination may have a role in substrate protein stability, conformation and interactome profile. Interestingly, the polyubiquitin assembly may also contain branched chains with different types of ubiquitin linkage [12,65].

### 3.2. The Opposite Effect of Ubiquitination: The Role of Deubiquitinases

Ubiquitination is a reversible reaction. Approximately 100 deubiquitinating enzymes (DUBs) play a role in this process in mammalian cells (Figure 2) [68]. DUBs are classified into six families: “ubiquitin C-terminal hydrolases” (UCHs), “ubiquitin-specific proteases” (USPs), “ovarian tumor proteases” (OTUs), Josephins and JAB1/MPN/MOV34 metalloenzymes, and “motif interacting with Ub-containing novel DUB family” (MINDY) [69]. There are three possible mechanisms by which DUBs can remove ubiquitin modifications: 1. endo-cleavage happens between the ubiquitin moieties, 2. exo-cleavage affects to the distal ubiquitin moiety and 3. the base-cleavage removes the whole ubiquitin chain from the substrate. Thus, the different DUBs, which have distinct specificities for linkage types of ubiquitination, can control the removal of ubiquitin chains from several types of protein substrates [68,69,70,71].

## 4. The Activity of Deubiquitinating Enzymes in Autophagic Mechanisms

In the second part of this review we gave a detailed overview about the mechanistic nature of lysosome-dependent self-degradative processes. Now, we will summarize the role of the most important DUBs in certain autophagic processes (Figure 1).

### 4.1. DUBs in Macroautophagy

The ubiquitination system, as well as deubiquitinating enzymes, is strongly involved in many aspects of macroautophagy, which was summarized in several review articles by Grumati and Jacomin [72,73]. The ubiquitin-proteasome system (UPS) has a main role in the ubiquitination and degradation of autophagy regulator proteins, which may directly affect autophagic flux. Several autophagy regulators, such as LC3 family members, with ubiquitin tags may also influence the regulation of autophagy via effecting protein folding and interactomes. For instance, the recently identified mechanism clearly shows that autophagy is negatively regulated by the ubiquitin activating protein Ubp6, and its interactor partner Birc6 ubiquitin ligase, via the polyubiquitination and designation for proteasomal degradation of the key macroautophagic factor LC3 (Atg8) [74]. However, the relevant DUBs, which have an opposite effect for LC3 stabilization, have not yet been identified. Moreover, the Lys33-linked ubiquitin chains can interact with the p62 autophagic receptor, and this interaction at the surface of autophagosomes is necessary for autophagosome formation [75].

The early step of macroautophagic machinery requires double-membrane-bound phagophore emergence in the cytoplasm and autophagosome formation. The main regulating factor of phagophore formation is the mammalian target of rapamycin (mTOR) which is negatively regulated by DEPTOR inhibiting the kinase activity of mTOR. In normal conditions DEPTOR is degraded continuously by the ubiquitin-proteasome system. During nutrient deprivation DEPTOR is stabilized by Otub1 deubiquitinase, which is the prerequisite of mTOR inactivation and contributes to the assembly of the Atg1/ULK1 complex (Atg1/ULK1, Atg13, Atg101), which is required for phagophore formation [73]. Importantly, the key member of this complex, Atg1/ULK1, is also positively regulated by Usp20 via the deubiquitination and stabilization of the Atg1/ULK1 protein. Atg6/Beclin1 activity, which is initialized and stabilized by Usp10 and Usp13 is necessary for expansion of the phagophore membrane [73]. Moreover, Usp33 also positively regulates Atg6/Beclin1 via the deubiquitination of its positive regulator protein RALB, by which Usp33 promotes the interaction of these proteins, thereby stimulating phagophore membrane growth. The negative regulation of Atg6/Beclin1 is materialized by the activity of Usp14 via the cleaving of K63-linked polyubiquitin chains from Atg6/Beclin1 rather than K48-linked chains, which designates this protein for proteasomal degradation [73]. Moreover, the positive transcriptional regulation of Atg6/Beclin1 and its binding partner Atg14L by the nucleolar localized DUB Usp36 is also necessary for autophagosome formation and Parkin-mediated mitophagy. Usp36 may have a role in the deubiquitination of the transcriptionally active chromatin marker histone 2B (H2B), which contributes to the positive transcriptional regulation of Atg6/Beclin1 and Atg14L [76]. Moreover, the aggregated proteins, intracellular bacteria and damaged mitochondria tagged with ubiquitin chains can be brought to the autophagosomal membrane via an autophagy receptor [77]. Interestingly, the selective macroautophagy also requires the contribution of DUBs for their normal mechanism. For example, the Parkin mediated macromitophagy (selective degradation of damaged mitochondria by macroautophagy) is negatively regulated by the deubiquitination activity of Usp30 on the mitochondrial outer membrane proteins [78,79,80]. Moreover, Usp8 facilitates macromitophagy by deubiquitination and stabilization of Parkin E3 ubiquitin ligase [73]. In the yeast model system, the operation of Ubp3 is important for the selective autophagic degradation of 60S ribosomes (macroribophagy). Interestingly, the deubiquitination of these ribosomal subunits by Ubp3 designates for selective macroautophagic degradation of these macromolecular complexes [81].

Autophagosome-lysosome fusion is a prerequisite of the autolysosome formation and the cytoplasmic cargo degradation. Kim et al. recently identified that Usp14 is an essential factor for autophagosome-lysosome fusion via the rescue of the microtubule-associated protein Tau (MAPT) from proteasomal degradation. Importantly, this protein has a role in the maintenance of microtubular dynamics and, thereby, in membrane fusion mechanisms, such as autophagosome-lysosome fusion [82,83]. Moreover, Usp14 may also be necessary to evade UVRAG (UV radiation resistance associated gene) from proteasomal degradation by its deubiquitination [82]. It is important to note that the UVRAG containing Vps34 kinase complex is required for lysosome maturation via the delivery of lysosomal hydrolases and membrane proteins from the Golgi to the lysosomal compartment [54,84].

Autophagic degradation also strongly depends on the activity of the v-ATP-ase proton pump and lysosomal hydrolases such as cathepsins [54,85,86]. The positive transcriptional regulation of cathepsin L (CTSL) by the activity of COP9 signalosome subunit 6 (CSN6) deubiquitinase was identified in cervical cancer cells [87]. Importantly, yeast orthologs of the above-mentioned DUBs also may contribute to the Cvt pathway in yeast, but little data is available on this.

### 4.2. Deubiquitinases in Microautophagy and Endosomal Microautophagy

Lysosomal and endosomal uptake of the neighboring cytosolic material is an important autophagic process as well. Usp8 (also known as UBPY) has an essential role in the deubiquitination of endocytosed membrane proteins prior to their lysosomal delivery and degradation, and is also required for multivesicular endosome (MVE) formation via its interaction with ESCRT proteins, such as ESCRT-0, the interplay of which leads to the stabilization of ESCRT machinery and multivesicular body formation [88,89,90,91]. Importantly, the ESCRT complex is also necessary for lysosomal/vacuolar microautophagy [36,38]. Based on the mechanistic similarity of microautophagy and endosomal microautophagy, it is possible that Usp8 (and his high scored orthologue in yeast Ubp5) also may have a functional role in lysosomal microautophagic process. Other endosomal deubiquitinase is the associated molecule with the SH3 domain of STAM (AMSH), which is necessary in the normal endosomal maturation processes. AMSH is able to cleave K48- and K63-linked polyubiquitin chains on the target proteins, such as ESCRT-0, which leads to the stabilization and activity alteration of these targets [92]. Importantly, the stabilized ESCRT proteins are the prerequisites for multivesicular endosome formation and lysosomal microautophagy [36,89,91]. The loss of function of AMSH and Usp8 causes a similar phenotype: accumulation of endocytic proteins in the endosomal compartments [90,92]. Taken together, the cooperation of AMSH and Usp8 endosomal DUBs is necessary for multivesicular endosome formation and endocytic cargo degradation.

### 4.3. The Possible Role of Other DUBs in Autophagy-Like Vesicular Trafficking Mechanisms

Several lysosome-dependent self-degradative processes use vesicular trafficking machinery to fuse the transported cargo-containing vesicle with the lysosome/vacuole.

Crinophagy is often used by several types of gland cells for the removal of unsecreted secretory granules from the cytoplasm. During this process the obsolete secretory vesicles directly fuse with late endosomes/lysosomes for the fast elimination of the secretory cargo [41,42]. Crinophagy is the least known autophagic process, but the molecules which coordinate the direct fusion of secretory granules and lysosomes were recently identified. For example, Rab7, a small GTPase occurring often in lysosome-dependent vesicular trafficking processes, is also necessary for crinophagy [27,43,93]. Our laboratory earlier showed that Rab7 is present on the surface of glue containing secretory granules before their fusion with late endosomes and lysosomes in *Drosophila* late-larval salivary glands. It is possible that Rab7 designates the obsolete secretory granules for degradation as a HOPS recruiting factor [43]. Interestingly, Sapmaz et al. recently identified that the late endosomal deubiquitinase Usp32 is required for the positive regulation of Rab7 recycling and recruitment to the late endosomal membranes. Moreover, the deubiquitination of Rab7 by Usp32 is necessary for transporting late endosomes to the lysosomal compartment [94]. Based on these results, it is possible that all Rab7 (and Ypt7 in yeast) dependent autophagic mechanisms, such as the MVE-lysosome fusion, autolysosome and crinosome formation, Cvt, ALP and the Vid pathway may also require the activity of Usp32, or its high scored yeast orthologue Ubp12.

Doa4 is the most commonly used DUB in vesicular trafficking processes by yeast cells. For example, in the vRed pathway, which is responsible for the selective degradation and recycling of the lysine efflux transporter (Ypq1), the influx Zn^2+^ transporter (Cot1) and the Zn^2+^ channel (Zrt3) molecules from the vacuolar membrane [15,16]. These transporter and channel molecules are ubiquitinylated by E3 ubiquitin ligases (Rsp5 and Tul1). Importantly, the Doa4 depleted cells showed highly ubiquitinated Ypq1 molecules on the vRed pathway. In conclusion, Doa4 may have a counter effect in the regulation of vRed and similar mechanisms, which may contribute to several monomer recycling pathways [15,16].

During the vacuolar import and degradation (Vid) pathway the unnecessary gluconeogenic enzymes are packed into small vesicles, which then directly fuse with the vacuole in yeast cells after glucose readdition [55]. The Vid pathway also requires Ypt7, which raises the possibility that Ubp12, the potential Ypt7 regulatory DUB in the yeast—based on the Usp32 data [94]—is also necessary for Vid vesicle-vacuole fusion [59]. Moreover, Wolters et al. recently evinced a possible regulatory connection between Vid27 (which is required for Vid vesicle clusters formation during the Vid pathway) and Doa4 molecules. Imaginably, Vid27 may act as a cofactor, which interacts with other DUBs partially redundant with Doa4 for its activation [95,96].

Importantly, vesicular trafficking mechanisms also require normal microtubule dynamics and motor proteins. Several articles show that Cylindromatosis (Cyld), a DUB, has an essential role in the regulation of microtubule dynamics. For instance, Gao et al. identified that, Cyld interacts with tubulins, and the activity of this DUB is required for microtubule assembly and maintaining its stability. Consequently, it is possible that Cyld is also necessary for several autophagic processes, which use vesicular trafficking mechanisms [83,97,98,99,100].

Taken together, lysosome dependent self-degradative pathways are richly controlled by several types of deubiquitinases, thereby, the latter have an essential role in the regulation of these autophagic processes (Figure 1). These relevant DUBs are summarized in Table 1.

## 5. Conclusions and Future Perspectives

Protein ubiquitination is a multi-functional reversible post-translational modification that affects all cellular processes. Over the past decade there have been considerable advances in the understanding of the function of DUBs, their mechanism of action and regulation. Recently, there has been an increasing body of evidence attesting that ubiquitination plays a crucial role in regulating various forms of autophagy, and DUBs interfere at several steps in these.

Autophagic processes are essential in maintaining normal cellular homeostasis via degrading and recycling unnecessary or damaged self-material from the cytoplasm. In this review we summarized the mechanism and molecular background of the known lysosome dependent self-degradative pathways. Autophagy has a colorful appearance in cells depending on the type of degraded cellular material [1]. Interestingly, ubiquitin, as a posttranslational signaling molecule, has a multiple role in the regulation of these processes [101]. Importantly, deubiquitinases may have several possible effects in the regulation of autophagic mechanisms.

1. *Stabilization/destabilization of autophagic substrates*. Ubiquitination and deubiquitination of autophagy substrates, as well as components of the autophagic machinery, are critical regulatory mechanisms of autophagy. The deubiquitination of these factors often causes its stabilization and contributes to several types of autophagy which take place. For instance, the activity of AMSH and Usp8 is necessary for the rescue of ESCRT proteins from proteasomal degradation [89,91,92]. Moreover, Usp8 also has a role in the stabilization of the macromitophagic key regulator, Parkin [73]. Thereby, Usp8 contributes to the macro- and microautophagic processes, too. It is important to note that Usp14 can stabilize the microtubule-associated protein Tau (MAPT) and UVRAG, the activity of which is necessary for all lysosomal fusion and degradation events [73]. On the other hand, the operation of Usp14 may cause the destabilization of Atg6/Beclin1 by removing K63-linked polyubiquitin chains rather than K48-linked chains from this substrate, so this DUB may obstruct the assembly of autophagosomes [82]. Interestingly, the deletion of ubiquitin from the 60S ribosomes by Ubp3 designates these macromolecules for their selective macroautophagic degradation (macroribophagy) [81]. Nota bene, the removal of ubiquitin patterns from substrates or autophagy proteins by DUBs is also essential for the regulation of these pathways.

2. *Regulation of protein localization*. Deubiquitination also has an effect on the autophagic protein intracellular localization. For example, Usp32 deubiquitinates the late endosomal marker Rab7, which is important for the recruitment of this protein to the late endosomes, and is also necessary in these protein recycling mechanisms [94]. Imaginably, this Usp32 dependent regulation of Rab7 may be essential in all lysosomal fusion events, such as autolysosome and crinosome formation as well as MVE-lysosome fusion. Importantly, Ubp12, which is a high scored orthologue of Usp32 in yeast, may have a role in “exotic” autophagic mechanisms, such as vRed-, Cvt-, ALP- and Vid vesicle-vacuole fusion events.

3. *Transcriptional regulation of the autophagic factors* are also regulated by several DUBs. For instance, Usp36 (localized in the nucleolus) has an essential role in the enhancing of the Atg6/Beclin1 and Atg14L expression levels. These proteins are required for the formation of autophagosomes, so the deubiquitinase activity of Usp36 indirectly facilitates autophagosome growth and thereby macroautophagy, especially macromitophagy [76]. On the other hand, the expression level of the lysosomal hydrolase Cathepsin-L (CTSL) is positively regulated by CSN6 deubiquitinase in cervical cancer cells [87]. It seems likely that CSN6 has a main contribution in the normal lysosomal maturation process, which is a prerequisite for the proper lysosomal degradation of several cytoplasmic and membrane bound substrates.

4. *Facilitation of microtubule assembly and maintaining its dynamics* took place by the activity of Cyld [97,99]. Importantly, the normal microtubule dynamics may also be essential for autophagy-like vesicular trafficking mechanisms, such as MVE-, autophagosome-, secretory granule-lysosome fusion and vRed-, Cvt- ALP- and Vid vesicle-vacuole fusion [83].

Deregulation in both autophagy and ubiquitination/deubiquitination processes has been linked to many pathological processes such as diabetes, neurodegenerative diseases, cancer onset and progression, accelerated aging mechanisms and different kinds of viral or bacterial infections. Consequently, autophagic processes, and also relevant DUBs, have high medical relevance [102]. The investigation of the connection between lysosome-dependent degradative pathways and their ubiquitin-mediated regulation is necessary for a better understanding of the pathological background of several autophagy connected disorders. Therefore, it is very important to study further the role of deubiquitinases in several types of lysosome-dependent self-degradative mechanisms, which may provide the opportunity to develop new therapies and drugs for more efficient treatment of autophagy related disorders.

## Figures and Tables

**Table 1:** Summary of the relevant deubiquitinases and their effects in several autophagic processes.

Deubiquitinase	Autophagy Type	Targets	Effects	References
AMSH	Microautophagy and endosomal microautophagy	ESCRT-0	Stabilization of ESCRT proteins, MVE formation	[91,92]
CSN6	Lysosomal degradation	Cathepsin-L (CTSL)	Positive transcriptional regulation of CTSL	[87]
Cyld	Autolysosome and crinosome formation, MVE-lysosome fusion, vRed, Cvt, ALP, Vid pathways	α- and β-tubulin	Microtubule assembly and maintaining microtubule dynamics	Data available about the regulation of microtubules [83,97,98,99,100]
Doa4	vRed and Vid pathways	Ypq1, Cot1, Zrt3, Vid27?	Opposite effect of Rsp5/Tul1 mediated degradation of Ypq1, Cot1, Zrt3, possible binding partner of Vid27	[15,16,95]
Otub1	Macroautophagy	DEPTOR	Stabilizes DEPTOR which inhibits mTOR, macroautophagy induction	[73]
Ubp3	Macroribophagy	60S ribosomes	Positive regulation of 60S ribosome macroautophagic degradation by its deubiquitination	[81]
Usp8/Ubp5	Microautophagy and endosomal microautophagy, macromitophagy	ESCRT-0, Parkin	Stabilization of ESCRT proteins, MVE formation, Parkin stabilization	Usp8 data only available [73,88,89,90,91]
Usp10	Macroautophagy	Atg6/Beclin1	Activation and stabilization of Atg6/Beclin1, positive regulation of autophagosome formation	[73]
Usp13	Macroautophagy	Atg6/Beclin1	Activation and stabilization of Atg6/Beclin1, positive regulation of autophagosome formation	[73]
Usp14	Macroautophagy	Atg6/Beclin1, MAPT, UVRAG	Destabilizes Atg6/Beclin1, rescues MAPT and UVRAG from proteasomal degradation	[73,82]
Usp20	Macroautophagy	Atg1/Ulk1	Positive effect on macroautophagy by stabilization of Atg1/Ulk1	[73]
Usp30	Macromitophagy	Mitochondrial surface proteins	Opposite effect of Parkin mediated macromitophagy	[78,79,80]
Usp32/Ubp12	Autolysosome and crinosome formation, MVE-lysosome fusion, vRed, Cvt, ALP, Vid pathways	Rab7/Ypt7	Positive regulation of Rab7/Ypt7 recycling and recruitment to the membrane of late endosomal compartment	Usp32 data about late endosomal effect only available [94]
Usp33	Macroautophagy	RALB	Stabilizes RALB, the Atg6/Beclin1 interacting protein, positively regulates autophagosome formation	[73]
Usp36	Macroautophagy, macromitophagy	direct effect on H2B, indirect effect on Atg6/Beclin1 and Atg14L	Positive transcriptional regulation of Atg6/Beclin and Atg14L	[76]
?	Macroautophagy	LC3/Atg8	LC3/Atg8 stabilization, opposite effect of Ubp6 and Birc6	[74]
